# *Pseudomonas* Phage Banzai: Genomic and Functional Analysis of Novel *Pbunavirus* with Lytic Activity Against *Pseudomonas aeruginosa*

**DOI:** 10.3390/v17081088

**Published:** 2025-08-06

**Authors:** Andrei V. Chaplin, Nina N. Sykilinda, George A. Skvortsov, Konstantin S. Troshin, Anna A. Vasilyeva, Sofia A. Shuraleva, Artem A. Malkov, Vladislav S. Simonov, Boris A. Efimov, Lyudmila I. Kafarskaia, Konstantin A. Miroshnikov, Anna A. Kuznetsova, Peter V. Evseev

**Affiliations:** 1 Pirogov Russian National Research Medical University, Ostrovityanova 1, 117997 Moscow, Russia; okolomedik@gmail.com (A.V.C.); galaxy22242500@mail.ru (G.A.S.); konstantinetr@gmail.com (K.S.T.); annavasilyeva.bioscience@gmail.com (A.A.V.); shuralyova_sa@rsmu.ru (S.A.S.); xkairyx@gmail.com (A.A.M.); propeltidii@gmail.com (V.S.S.); efimov_ba@mail.ru (B.A.E.); likmed@mail.ru (L.I.K.); kuznetsova.anna7377@gmail.com (A.A.K.); 2 Shemyakin-Ovchinnikov Institute of Bioorganic Chemistry, Russian Academy of Sciences, Miklukho-Maklaya Str. 16/10, 117997 Moscow, Russia; sykilinda@mail.ru (N.N.S.); kmi@bk.ru (K.A.M.)

**Keywords:** phage, *Pbunavirus*, *Lindbergviridae*, phage evolution, *Pseudomonas aeruginosa*, phage genomics, phage therapy, antimicrobial resistance

## Abstract

Antibiotic-resistant *Pseudomonas aeruginosa* presents a critical global health challenge, particularly in hospital-acquired infections. Bacteriophages offer a promising therapeutic avenue due to their ability to target and lyse resistant strains. This study characterizes *Pseudomonas* phage Banzai, a newly isolated *Pbunavirus* (family *Lindbergviridae*) with lytic activity against multiple *P. aeruginosa* isolates, including multidrug-resistant strains. Genomic analysis revealed a 66,189 bp genome, lacking antibiotic resistance or virulence factors, and suggested a headful packaging mechanism and the presence of a bidirectional component in the replication. In vivo experiments using *Galleria mellonella* showed therapeutic potential, significantly improving larval survival (87% at 24 h). Host range analysis revealed activity against 13 of 30 *P. aeruginosa* isolates, including members of O1, O3, O5 and O6 in silico predicted serogroups. Phylogenomic analyses place phage Banzai within the genus *Pbunavirus*, sharing 94.8% intergenomic similarity with its closest relatives, supporting its classification as a novel species. These findings highlight phage Banzai as a potential candidate for phage therapy, demonstrating genomic stability, a strictly lytic lifestyle, and in vivo efficacy.

## 1. Introduction

Bacteriophages (phages), bacterial viruses that are specific to bacteria, are a major component of the biosphere, possibly constituting the most numerous biological entities on the planet. Their significance extends to the dynamic regulation of microbial communities in various settings, from pristine natural environments to the complex human microbiota [1]. The ability of bacteriophages to destroy bacteria attracted attention soon after their discovery at the beginning of the twentieth century, independently by Twort in 1915 and d’Herelle in 1917 [2]. Furthermore, bacteriophages present compelling opportunities for evolutionary biology studies, contributing significantly to our understanding of bacterial evolution and offering valuable tools for the deeper exploration of fundamental molecular and biophysical inquiries [3,4]. Driven by the escalating threat of antibiotic resistance, bacteriophages have experienced a renaissance in interest as potential therapeutic agents for treating infections caused by multi-drug resistant organisms [5].

Hospital-acquired (nosocomial) infections caused by antibiotic-resistant microorganisms have become a recognized global health concern within the modern healthcare system. *Pseudomonas aeruginosa*, a Gram-negative bacterium capable of causing severe opportunistic infections, is of particular concern. The increasing antibiotic resistance exhibited by this pathogen warrants significant attention, leading to its inclusion in the ESKAPE group—a collection of the most problematic resistant bacteria [6]. *P. aeruginosa* produces a multitude of virulence factors exacerbating inflammatory processes [7,8]. This bacterium is ubiquitous in the environment, as well as prevalent in healthcare settings, readily colonizing human mucosal surfaces [9,10]. *P. aeruginosa* infections in immunocompromised patients, or in the presence of multi-drug-resistant strains, can result in the development of severe and life-threatening conditions, including ventilator-associated pneumonia, otitis, urinary tract infections, sepsis, and burn wound infections [7,9,10,11,12,13,14,15]. The mortality rate associated with these infections can reach values approaching 20–40% [16,17], underscoring the urgent need for effective therapeutic interventions and the development of novel antibacterial agents. Given the limited availability of effective antimicrobial drugs, phage therapy has emerged as a promising approach. Studies conducted in animal models and clinical trials have demonstrated that phages, either as a monotherapy or in combination with antibiotics, can successfully suppress multi-drug resistant (MDR) *P. aeruginosa* strains, thus opening new avenues for the treatment of these recalcitrant infections [18,19,20,21,22].

Bacteriophages are generally categorized based on two distinct lifestyles: temperate and lytic. Temperate phages possess the capacity to enter the lysogenic cycle, a process characterized by the integration of phage genomic DNA into the host chromosome. This integrated phage DNA, known as a prophage, replicates in synchrony with the host’s genomic DNA. In contrast, lytic phages are unable to engage in lysogeny and propagate solely through host cell lysis. Conventional phage therapy protocols typically advocate for the exclusive use of lytic phages, whose genomes are devoid of genes encoding potential virulence factors or antibiotic resistance determinants [23].

Bacteriophages infecting *Pseudomonas* species are extensively studied. As of June 2025, GenBank’s phage database contained 53,047 records, with 2531 specifically identified as *Pseudomonas* phages. Among these, 2154 sequences include laboratory host or host attributes, and a substantial majority (1426) target *P. aeruginosa* strains.

*Caudoviricetes* phages infecting *P. aeruginosa* are represented across approximately one hundred genera. These include phages classified directly under the class *Caudoviricetes*, as well as those assigned to the order *Autographivirales* and various families/subfamilies. Additionally, genomic evidence indicates that dozens of unclassified temperate *Pseudomonas* phages likely constitute novel genus- or higher-level taxa [24].

Lytic tailed phages targeting *P. aeruginosa*, many with therapeutic potential, are remarkably abundant and diverse. Key groups encompass the order *Autographivirales* (elevated from family rank in 2024 and ratified by the International Committee on Taxonomy of Viruses in 2025), the families *Chimalliviridae*, *Mesyanzhinovviridae*, *Schitoviridae*, and *Vandenendeviridae*, and the subfamily *Queuovirinae* [20,25]. The genus *Pbunavirus*, assigned to the newly established family *Lindbergviridae* (ICTV-ratified February 2025), comprises the most extensively sequenced group of *Pseudomonas*-targeting phages, with 227 genomes currently deposited in GenBank. Pbunaviruses, also known as PB1-like phages, also lead in clinical phage therapy research [20], demonstrating considerable therapeutic promise [26,27,28,29] due to their high lytic efficacy. Phage PA_LZ01 significantly reduced the bacterial load and inflammatory response in an intraperitoneal infection mouse model [30]. A nebulized three-phage cocktail including one *Pbunavirus* decreased *P. aeruginosa* sputum density in adult cystic fibrosis patients [31]. Notably, CryoEM analysis of *Pbunavirus E217* and *Pbunavirus Pa193* has provided valuable insights into virion architecture and early infection mechanisms [32,33]. However, comprehensive phylogenomic studies essential for understanding this group’s evolutionary trajectories remain notably absent.

The present study conducts a comprehensive analysis of the newly isolated *Pbunavirus* phage Banzai infecting *Pseudomonas*. The name reflects both a traditional Japanese exclamation and relates to the “B-one” fragment in the name of the PB1-like group of phages. We characterize its fundamental biological properties and lytic efficacy using *Galleria mellonella* larvae as an in vivo model offering significant translational advantages. Distinct from mammalian models, *G. mellonella* entails no ethical restrictions, requires no feeding during experiments, and permits precise pathogen/antimicrobial dosing via direct injection, thereby minimizing experimental variability. Critically, results are rapidly obtained within 24–48 h, and infections proceed at 37 °C, mirroring human physiology. This model’s utility for phage infectivity and antibacterial activity assessment is well-documented [34]. Complementing these assays, we perform exhaustive genomic and proteomic characterization of phage Banzai, integrating phylogenomic reconstructions, structural modeling of predicted proteins, and functional annotation to propose evolutionary hypotheses for *Pbunavirus* diversification and host adaptation mechanisms.

## 2. Materials and Methods

### 2.1. Phage Isolation and Purification

*Pseudomonas aeruginosa* PAO1, obtained from the collection of Professor V.N. Krylov (I.I. Mechnikov Research Institute of Vaccines and Serums, Moscow), served as the host strain for bacteriophage propagation. To facilitate phage enrichment, a 3 mL water sample obtained from Yuryevsky pond, Voronovo district, Troitsky administrative district, Moscow region (coordinates 55°19′26″ N, 37°6′23″ E) was supplemented with 1 mL of 4× Lysogeny broth (LB) (Becton-Dickinson, Franklin Lakes, NJ, USA) and 40 µL of an overnight culture of *P. aeruginosa* PAO1. Following an 18 h incubation period at 37 °C, chloroform (Aldosa, Moscow, Russia) was introduced to a final concentration of 0.5% (*v*/*v*), and the mixture was incubated for 4 h at 4 °C, subsequently followed by centrifugation. The presence of bacteriophages was verified utilizing a double-layer agar technique [35], employing a soft agar overlay containing 0.75% agar. Serial dilutions of the bacteriophage-containing supernatant were plated onto a *P. aeruginosa* lawn, and a single plaque was selected following incubation for 18–24 h for subsequent propagation. To propagate the bacteriophage derived from a single plaque, *P. aeruginosa* PAO1 was infected in liquid culture at 37 °C. Upon complete lysis, the bacteriophage lysate was subjected to chloroform treatment followed by centrifugation at 8000× *g* and precipitation using polyethylene glycol (10%) (NeoFroxx, Einhausen, Germany)/NaCl (1M), followed by centrifugation at 8000× *g* (Nuve NF 1200 R, NÜVE, Ankara, Turkey) and resuspension in SM buffer (50 mM Tris-HCl, 100 mM NaCl, 8 mM MgSO_4_, pH 7.5). The final bacteriophage stock exhibited a titer of approximately 10^10^ PFU/mL and was stored at 4 °C until further use.

### 2.2. Experimental Procedures: Phage Adsorption and One-Step Growth

The determination of the phage adsorption rate was performed according to the method outlined in reference [36], with minor modifications. Briefly, 10 mL of *P. aeruginosa* PAO1 were cultivated in LB medium at 37 °C with agitation until reaching the exponential growth phase (OD600~0.4). Subsequently, bacteriophage Banzai was added at a multiplicity of infection (MOI) of 0.001, and the resulting mixture was subjected to vortexing. Aliquots (100 μL) were withdrawn at time points 0, 1, 2, 3, 4, 5, 10, and 15 min post-phage addition, treated with chloroform, and maintained on ice for 15 min. Following centrifugation at 10,000× *g*, the supernatant was subjected to titration to quantify the number of unadsorbed bacteriophages at each designated time point. The adsorption curve was generated by plotting the ratio of unadsorbed bacteriophage particles (P) to the initial bacteriophage titer (P0). This experiment was conducted in triplicate.

To determine the single-cycle burst size, a 20 mL culture of *P. aeruginosa* PAO1 at OD600~0.4 was centrifuged at 8000× *g*, resuspended in 0.5 mL of LB, and infected with 100 µL of bacteriophage at an MOI of ~0.01, followed by a 10 min incubation period. Unadsorbed bacteriophages were subsequently removed via centrifugation for 2 min at 13,000× *g*, and the resulting pellet was resuspended in 10 mL of LB. The suspension was incubated in 50 mL Eppendorf tubes at 37 °C on a shaker for 120 min at 170 rpm (ES-20 orbital shaker, Biosan, Hangzhou, China), with 100 µL samples collected at the initiation of the experiment and at 10 min intervals for up to 120 min post-initiation. This experiment was performed in triplicate. For PFU/mL quantification, the samples were subjected to titration, and 10 µL aliquots were spotted onto double-layer agar plates. Plaques were enumerated following a 24 h incubation period. Bacteriophage burst size was calculated as the ratio of the average titer at the plateau phase, post-lysis, to the total number of bacteriophage particles used to infect the bacterial cells at time zero (t0) [37].

### 2.3. Experimental Procedures: Phage Stability Under Different Conditions

Phage stability under various conditions was assessed following a modified protocol based on [38]. To determine temperature stability, a phage suspension was prepared in the SM buffer to a final concentration of 4.5 × 10^6^ PFU/mL. This suspension was then incubated at +30 °C, +40 °C, +50 °C, +60 °C, and +70 °C for 1 h using a TDB-120 dry block thermostat (Biosan, Riga, Latvia). To assess pH stability, SM buffer solution was adjusted with NaOH and HCl to pH values 3, 5, 7, 9 and 11. These solutions were added to the samples, achieving a final phage titer of 10^6^ PFU/mL, and incubated at 25 °C for 1 h. To determine UV resistance, high-titer phage samples (4.5 × 10^7^ PFU/mL) were exposed to a PL-S 9W/12/2p UV lamp (Philips, Amsterdam, The Netherlands) (280–315 nm) for 50 min. Aliquots of 50 µL were collected every 10 min into separate tubes. Chloroform sensitivity was evaluated by mixing phage solutions with chloroform to achieve final concentrations of 0%, 5%, 25%, 50%, and 75% (*v*/*v*), resulting in a final titer of 3 × 10^6^ PFU/mL. These mixtures were incubated at 37 °C for 30 min with shaking, according to [39]. Subsequently, the solutions were centrifuged at 10,000× *g* for 10 min, and the supernatant was collected. All solutions obtained were titrated using the LB top agar method. Following 24 h of cultivation, the titer in each sample was determined. All experiments were performed with three parallel repetitions.

### 2.4. Phage Host Range Determination

The lytic activity and host range of the bacteriophage were evaluated against a panel of 30 clinical and environmental *P. aeruginosa* isolates utilizing the double-layer agar assay. Clinical isolates, as described in reference [40], were obtained from the Russian Children’s Clinical Hospital. Overnight cultures of *P. aeruginosa* (200 μL) were combined with 3 mL of 0.75% soft agar and subsequently plated. Bacteriophage suspensions (~10^9^ PFU/mL) were then spotted onto the prepared bacterial lawns and incubated at 37 °C for 18–24 h. Following incubation, the plates were carefully examined to assess bacteriophage lytic activity and host specificity.

### 2.5. Transmission Electron Microscopy

The morphology of phage Banzai was visualized using transmission electron microscopy. Concentrated and purified phage samples were prepared by adsorption onto grids followed by negative staining with 1% uranyl acetate (pH 4.0). Images were acquired using a LEO Omega 912 AB electron microscope (Carl Zeiss SMT, Oberkochen, Germany).

### 2.6. MIC Analisys

In vitro activity of phage Banzai was assessed using the minimum inhibitory concentration (MIC) method. Briefly, a homogenized bacterial inoculum with an OD of 0.5 (McFarland standard; ~4.0 × 10^7^ CFU/mL) was prepared from an overnight culture. For each test well, 150 μL of 2× LB, 50 μL of phage Banzai, and 20 μL of the bacterial suspension were added. Each assay was performed in triplicate. To control for phage lysate sterility, 150 μL of nutrient medium was mixed with 50 μL of the phage preparation. A bacterial growth control was also included, consisting of 150 μL of LB with 20 μL of the bacterial inoculum. The plates were then incubated for 18 h at 37 °C.

### 2.7. Testing Phage Activity In Vivo Using Galleria mellonella Model

*G. mellonella* was ordered at a local vendor. Stored in the dark, at room temperature in a glass jar with ventilation openings. Larvae were used on the next day after obtaining. Inoculum was prepared according to the following protocol: *P. aeruginosa* was cultivated overnight in LB medium at 37 °C and 250 rpm. Overnight culture was diluted 1:100 into the fresh media and incubated subsequently again at 37 °C and 250 rpm until an OD 600 = 0.6–0.8 was reached. Then, using the serial dilution method, the final *P. aeruginosa* solution with a concentration of 1 × 10^5^ CFU/mL was made.

For larvae infection, *G. mellonella* larvae were divided into three groups, each group containing 15 larvae: the first treated with a 0.9% NaCl solution, the second treated with a *P. aeruginosa* solution, and the third treated with *P. aeruginosa* and a solution of phage Banzai.

The protocol used for infection is as follows:

1. Prepare 15 Petri dishes and label them accordingly (3 Petri dishes per group; each Petri dish will contain 5 larvae).
2. Prepare 15 tubes with 10 μL of 0.9% NaCl solution, 30 tubes with 5 μL of P. aeruginosa solution, and 15 tubes with 5 μL of phage Banzai (2 × 10^9^ PFU/mL concentration was used in this study).
3. Grab a larva randomly from the glass jar with the tweezers and put it on the Petri dish.
4. Now take the insulin syringe and fill it with the necessary solution, then apply the needle into the last proleg of the larva. Press the plunger to inject the volume. Gently remove the pressure and subsequently the needle from the larva.
5. Note: For the third group, firstly fill the syringe with 5 μL *P. aeruginosa* solution and then add 5 μL phage solution.
6. Place it in one of the previously prepared Petri dishes.
7. Repeat this procedure now for all larvae.
8. After these steps we will have for each group three Petri dishes with five larvae each.
9. Place the Petri dishes now into a cardboard box containing air holes and incubate the larvae in the dark at 37 °C.

Scoring of the infection is as follows:

1. Monitor the larvae after 24 and 48 h, respectively.
2. Evaluate the efficiency of the phage by scoring for alive and dead larvae.
3. Analyze the data to produce a survival curve, which reflects the killing activity of phage Banzai in vivo.

### 2.8. Bioinformatic Analyses

Reads were trimmed using fastp 0.24.0 [41] and assembled with Unicycler 0.5.0 [42]. Coding sequences were identified using Pharokka 1.7.5 [43]. Gene boundaries were manually curated. As the initially circular contig was obtained, the first genome position was set to the beginning of the terminase large subunit gene.

Functional annotation of the predicted genes was performed using a combination of sequence similarity and structure-based approaches. HHpred searches were performed against the PDB70_mmCIF70_30_Mar, PfamA-v37, UniProt-SwissProt-viral70_3_Nov_2021, and NCBI_Conserved_Domains(CD)_v3.19 databases [44]. Putative functions were assigned based on an HHpred probability of 95%. Foldseek searches [45] were performed using structures predicted by AlphaFold 3 [46] against PDB and AFDB-Swissprot databases, the structural alignments were manually curated. Default settings were used for all search engines. For the structural proteins studied using cryogenic electron microscopy in Pa193, previously published names were used [32]. The annotated genome was deposited in the NCBI GenBank database under accession number PV661111.

Phage termini and packaging mechanism were elucidated using PhageTerm 1.0.12 available on the Galaxy Pasteur server. Cumulative GC Skew weas calculated across the phage genome using the iRep package (gc_skew.py). To compare GC Skew within the genus, 45 representative *Pbunavirus* genomes were taken, one per species; four genomes with length < 63 kbp (due to partial sequencing or internal deletions) were discarded from this sample, as well as one genome with length 68.8 kbp (due to duplications or misassembly). Genome lengths of the remaining sequences were in the range from 64.1 kbp to 66.8 kbp. Starting positions within all these genomes were set to the beginning of the terminase large subunit gene.

Protein alignments were made using Muscle5 [47]. Phylogenetic analysis of the aligned viral sequences was performed using IQ-TREE 2.4.0 [48] with ModelFinder [49] to determine the optimal substitution model. Intergenomic comparisons of phages were conducted using the VIRIDIC web server [50].

Predicted 3D structures were visualized using UCSF ChimeraX [51]. Shannon entropy of a multiple sequence alignment column was used as a measure of amino acid variability [52].

In silico serogroup prediction of *P. aeruginosa* strains was performed using the pasty tool [53], implementing the locus database of Past 2.0 [54]. Defense systems were searched using DefenseFinder [55]; only complete systems (non-separate HMMer hits) were analyzed. Enrichment of defense system subtypes in phage-resistant or O11 strains was assessed using Fisher’s exact test with Benjamini–Hochberg correction for multiple comparisons, number of defense systems was compared using Student’s *t*-test. The significance level was set at 0.05 for statistical tests. Additionally, we performed a tBLASTn search for *P. aeruginosa* END nucleases described by Yee et al. [56], but no homologs with >20% identity were found in any strain.

## 3. Results

### 3.1. Latency Period and Burst Size Determination

**Figure 1:**
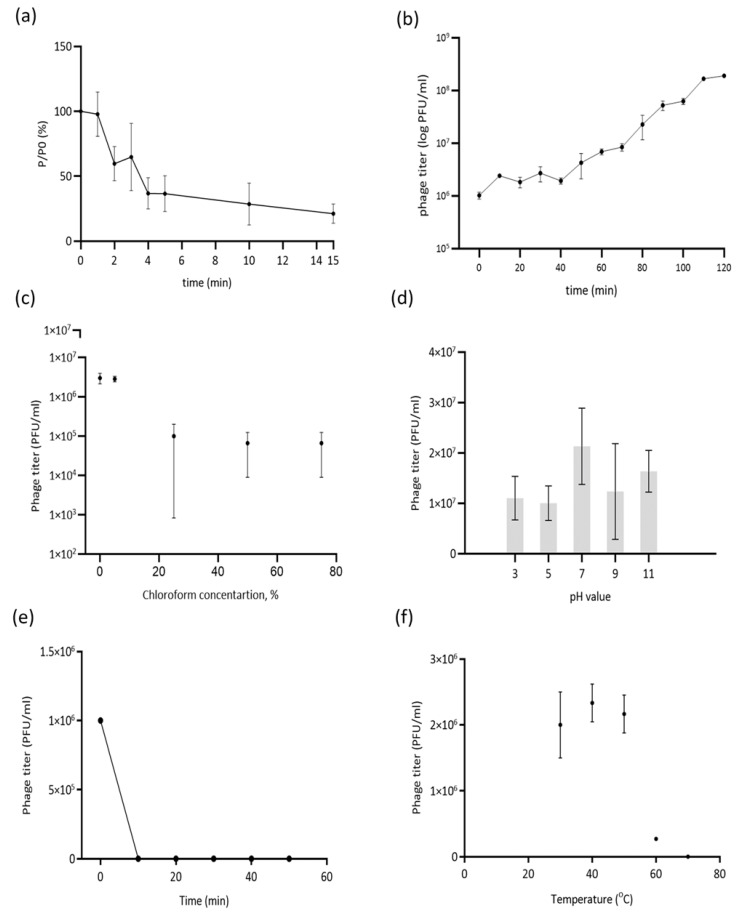
(**a**) Adsorption of phage Banzai at the surface of *P. aeruginosa* PAO1 in MOI = 0.001. (**b**) One-step growth curve of phage Banzai using *P. aeruginosa* PAO1 as the host strain in MOI = 0.01. Data points indicate the PFU/mL at different time points. (**c**) Chloroform treatment (0–75% *v*/*v*); (**d**) pH stress (pH 3–11 for 1 h); (**e**) UV irradiation (10–50 min); and (**f**) temperature stress (30 °C to 70 °C for 1 h). Data represent the mean of three independent replicates; error bars indicate standard deviation (SD).

About 63% of phage particles were adsorbed within 4 min, and phage was adsorbed almost completely (78%) only after 15 min (Figure 1a). According to the one-step growth curve, the latent period of Banzai phage was determined to be 50 min, followed by a rise period of 50 min, and the plateau was reached in 100 min after the start of the experiment. The estimated burst size was 104 ± 6 phage particles per infected cell (Figure 1b).

### 3.2. Phage Stability Under Different Conditions

After incubation in 25% chloroform solution, phage Banzai retained approximately 2% of its initial titer. The phage titer decreased in correlation with increasing chloroform concentration. (Figure 1c). The optimal pH value for phage Banzai is staying at 7. Exposure to pH values of 3, 5, 9 and 11 resulted in a partial loss of viability. (Figure 1d). Exposure to UV irradiation resulted in the complete inactivation of phage virions after 10 min (Figure 1e). The infectivity of phage Banzai after exposure to various temperatures for 1 h remained relatively stable between +30 °C and +50 °C. Specifically, the phage titer decreased by 55.6% at +30 °C and at +50 °C. However, exposure between +60 °C and +70 °C resulted in a decrease in titer at +60 °C by 94.0% and a complete loss of phage viability at +70 °C after 1 h of incubation (Figure 1f).

### 3.3. Phage Host Range

The host range of phage Banzai was qualitatively characterized by testing its lytic activity against 30 *Pseudomonas aeruginosa* isolates in a double-layer agar assay. This panel included 28 clinical isolates, the *P. aeruginosa* PAO1 type strain, and a laboratory-adapted PAO1 strain from the collection of Professor V.N. Krylov (Supplementary Table S1). The phage exhibited an average host range, with lytic activity observed against 13 of the 30 isolates. These susceptible isolates were found to represent sequence types ST12, ST200, ST231, ST242, ST254, ST274, ST358, ST395, ST549, ST2465 and ST3765. In silico serotyping of 27 *Pseudomonas* strains with available genomic sequences from this set demonstrated that Banzai lysed one O1 serogroup isolate, two of three O3 serogroup isolates, four of six O5 isolates and four of eight O6 isolates, but not the O2 isolate and none of the eight O11 serogroup isolates, indicating a broad spectrum of recognized O-specific antigens.

To elucidate the determinants of phage host range, we performed a genomic search for phage defense systems across the tested strains. In total, we identified 67 distinct defense system subtypes (using DefenseFinder nomenclature) within the 27 *Pseudomonas* genomes, with each strain harboring between 2 and 12 systems. No significant difference was found in the number of defense systems between sensitive and resistant strains.

Phage Banzai successfully lysed two or more strains encoding for defense systems of subtypes Olokun, CBASS_I, CAS_Class1-Subtype-I-E, RM_Type_I, Prithvi, PD-Lambda-5, Prometheus, Rst_HelicaseDUF2290, Erebus, RM_Type_II, PrrC, BstA, CAS_Class1-Subtype-I-F, Gabija, and Septu. These findings suggest that Banzai possesses the mechanisms to bypass (e.g., via DNA hypermodification) or inhibit these listed anti-phage systems.

Conversely, it is more challenging to determine which defense systems successfully counteract the phage infection. We found no statistically significant enrichment of any particular defense system subtype among the Banzai-resistant strains when compared to sensitive ones. This outcome can be explained by two main non-exclusive possibilities: the resistance is mediated by a multitude of rare defense systems, each protecting only a small subset of strains, or the predominant role of the O-specific antigen structure, which is the *Pbunavirus* receptor, in determining host range.

Focusing on the eight O11 strains, all of which exhibited resistance to phage Banzai, we found that their genomes were enriched in defense system subtypes AbiE, Prometheus, RM_Type_IIG, RM_Type_IIG_2, and RloC compared to non-O11 strains. However, none of these defense systems was universally present across all eight O11 genomes. This heterogeneity suggests that no individual system can fully account for their shared resistance, reinforcing the hypothesis that the O-specific antigen is the primary determinant.

### 3.4. MIC Analisys

After 18 h of incubation we received the following results of in vitro activity of phage: The MIC of phage Banzai was measured and resulted in 2.2 × 10^9^ PFU/mL. After that the MOI (multiplicity of infection) was calculated and resulted in 36 phage particles per bacterial cell.

### 3.5. Survival Curve of G. mellonella Treated with Phage Banzai

**Figure 2:**
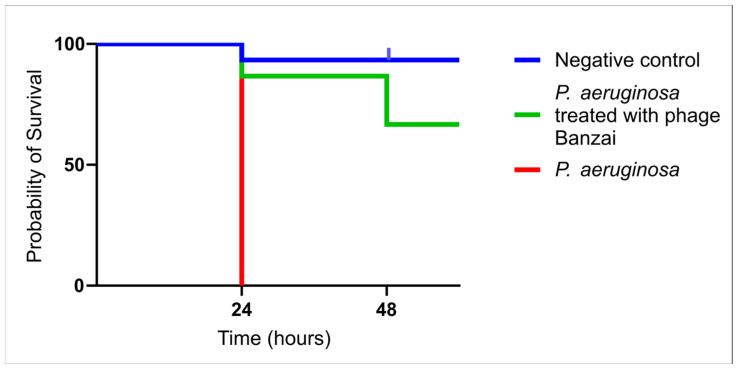
Survival curves of *Galleria mellonella* infected with *Pseudomonas aeruginosa* and treated with *Pseudomonas* phage Banzai.

To test the antimicrobial activity of the phage Banzai, we proposed an in vivo model with *G. mellonella* larvae, where larvae were treated with *P. aeruginosa* and with phage Banzai. The results showed significant improvement of larvae survival after 24 h with the treatment of phage Banzai (87%) and after 48 h (67%). The control group was treated with 0,9% NaCl solution and showed a 93% survival rate, while the group treated with *P. aeruginosa* showed a 100% mortality rate (Figure 2).

### 3.6. Phage Morphology

**Figure 3:**
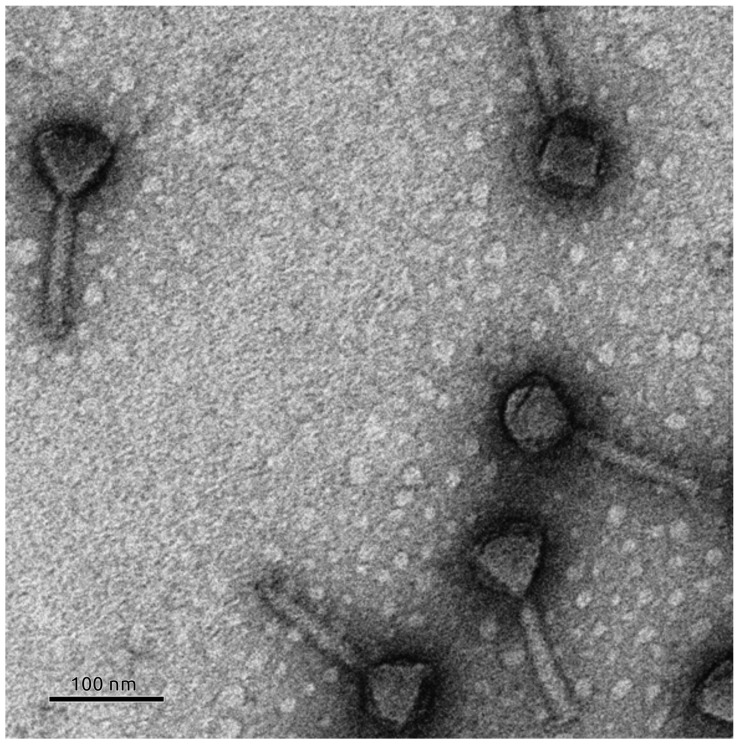
Electron microscopic image of phage Banzai particles. The scale bar is 100 nm.

Transmission electron microscopy revealed that phage Banzai exhibits a morphology characteristic of myoviruses, with an icosahedral head of approximately 70 nm in diameter measured from facet to facet and a contractable tail of ~130 nm in length (Figure 3).

### 3.7. Genome and Proteome Characterization of Phage Banzai

**Figure 4:**
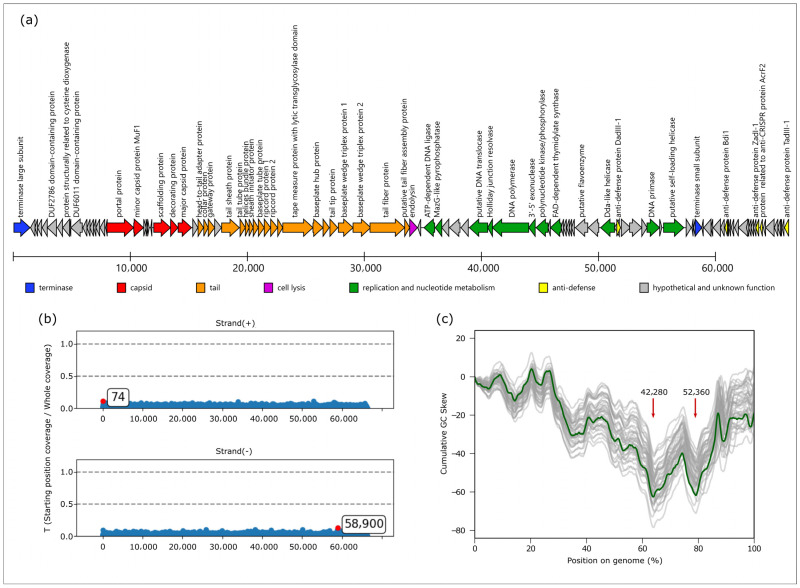
(**a**) Genome map of *Pseudomonas* phage Banzai. Each arrow represents one coding sequence. (**b**) Starting position coverage bias calculated using Phageterm. Positions of largest peaks are marked. (**c**) Cumulative GC skew of *Pseudomonas* phage Banzai (green) and 40 *Pbunavirus* genomes of other species (semitransparent gray). Local minima putatively close to replication origin(s) are shown with arrows, coordinates for the genome described are given.

The *Pseudomonas* phage Banzai genome comprises a double-stranded DNA molecule of 66,189 base pairs (NCBI Accession PV661111) and contains 105 predicted coding sequences (Figure 4a). GC content of the sequence is 55.5%. A BLAST search of the whole sequence against the NCBI core_nt database indicated a similarity to phages classified by the ICTV within the *Pbunavirus* genus, *Lindbergviridae* family. Analysis revealed the absence of any tRNA genes.

During de novo assembly of *Pseudomonas* phage Banzai, a circular contig was obtained, similarly to previous findings [26,57]. Analysis of read start coverage with PhageTerm indicated no peaks associated with coverage drops, which are characteristic for cohesive ends (because fragments crossing genome termini are originated only from contaminating circular DNA), nor twice the coverage region representing direct repeats [58]. Obtained results without significant peaks are most in line with the headful packaging mechanism forming a circularly permuted genome (Figure 4b).

Cumulative GC skew plots can provide a way of identifying origins of replication. In bidirectionally replicating DNA molecules (e.g., bacterial chromosomes), the lowest point in such a plot usually corresponds to the origin of replication and the highest point to the replication termination point, while in unidirectional phages, cumulative GC skew is monotonous [59,60]. In *Pseudomonas* phage Banzai, two minima are present, located in positions 42,280 and 52,360, and the general pattern indicates the presence of a bidirectional component in the replication (Figure 4c). Noteworthy, these points are located within the genome region encoding for replication-related proteins. Comparing GC skew in representative genomes of other *Pbunavirus* species, we observe that the most prominent minimum corresponds to position 42,280 in *Pseudomonas* phage Banzai, which is located within the DNA polymerase gene.

**Figure 5:**
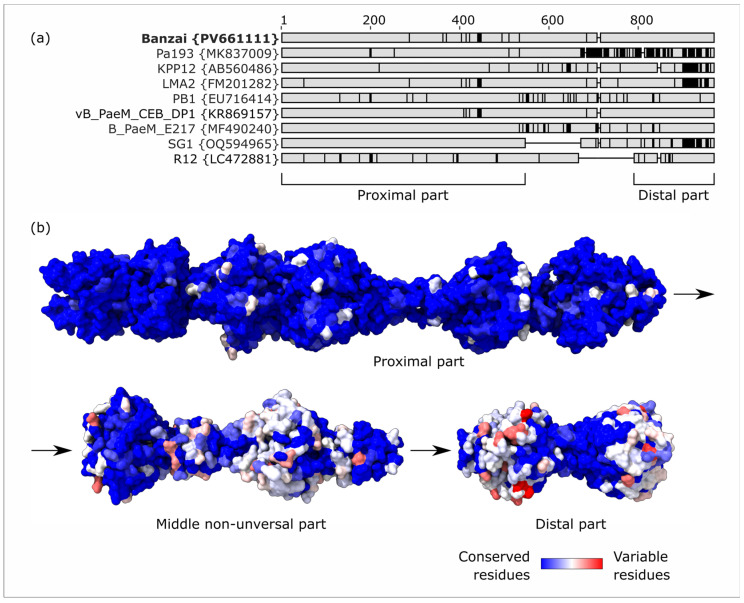
(**a**) Multiple sequence alignment of tail fiber proteins of several *Pbunavirus* phages. Vertical black lines, positions deviating from consensus; horizontal lines, gaps. (**b**) AlphaFold3 predicted the structure of three fragments of *Pseudomonas* phage Banzai tail fiber protein. Coloring represents Shannon entropy of related multiple sequence alignment columns based on 45 (proximal and distal part) or 43 (middle non-universal part, *Pbunavirus SG1* and *Pbunavirus RG12* excluded) genome sequences representing different *Pbunavirus* species.

Tail fibers of pbunaviruses are supposed to be responsible for cell attachment by binding *Pseudomonas* O-antigen. While we found the full-length tail fiber gene in all classified *Pbunavirus* species except for *Pbunavirus datas* (which has a massive deletion in the structural genes region), sequences of these proteins demonstrate prominent variability and mosaicism, with local drops of amino acid identity below 70% (an example is given in Figure 5a). The proximal part and distal tip are present in representative genomes of all species, while the middle part contains deletions in *Pbunavirus SG1* and *Pbunavirus RG12*. We found that the most variable parts of the tail fiber are three distal “knobs” (Figure 5b), and we hypothesize that they are responsible for recognition of different serotypes of O-antigen.

Neighboring genes encoding proteins whose structure was not elucidated using cryo-EM are also probably included in the virion structure or participate in morphogenesis. For example, protein PBanzai_019, the gene of which is located immediately downstream of the portal protein gene, possesses the Phage_Mu_F domain (PF04233.19), also designated as the MuF1 domain by Jamet et al. [61], similar to gp7 of *Bacillus* phage SPP1. These proteins are proposedly packed into capsid by interaction with the portal oligomer and subsequently delivered to the host at the beginning of infection [61,62].

**Figure 6:**
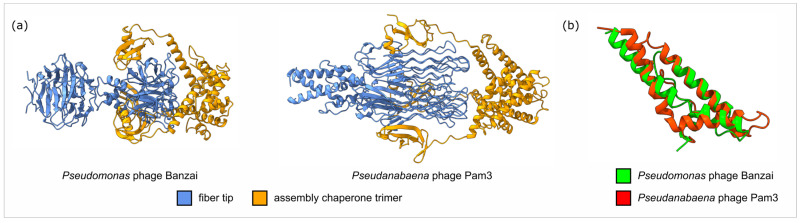
(**a**) AlphaFold3 predicted structure of the “distal part of tail fiber—tail fiber assembly chaperone trimer” complex compared with the structure of the related complex of cyanophage Pam3 (PDB: 7YPX) (**b**) Superimposition of C-terminal trimerization domain structures of assembly chaperone monomer.

Protein PBanzai_048, whose gene is located immediately downstream of the gene for tail fiber protein, represents a putative assembly chaperone. AlphaFold3 demonstrates its trimer-to-trimer binding with fiber (Figure 6a), with the globular N-terminal domain interacting with the distal tip and the C-terminal alpha-helical bundle mediating trimerization like previously revealed in cyanophage Pam3 gp25 [63]. C-terminal domains of these assembly chaperones demonstrated significant structural similarity (RMSD 3.0 Å) despite low amino acid identity (Figure 6b). A proteomic study of the related *Pseudomonas* phage Pa193 did not discover this protein in mature virions, unlike the ones in *Escherichia coli* phages Mu and P2, which directly participate in lipopolysaccharide binding [64].

The phage possesses an elaborate gene set encoding replication machinery, co-localized in a single region. The central role is played by DNA polymerase (PBanzai_058) and 3′-5′ proofreading exonuclease (PBanzai_059), homologous to DNA polymerase III subunits alpha and epsilon, respectively. The discontinuous synthesis of the lagging strand is facilitated by primase (PBanzai_075) and DNA ligase (PBanzai_051). The region encodes for three enzymes with ATP-dependent helicase/translocase activity. The first one (PBanzai_077) is homologous to hexameric self-loading helicases of staphylococcal phage-inducible chromosomal islands, which are able to recognize specific DNA sites and initiate DNA unwinding by themselves [65]. This protein is the prominent candidate for the replication initiation protein of bunyaviruses. The second one (PBanzai_070) is structurally related to the T4 phage monomeric SF1B helicase Dda. The last one (PBanzai_056) is mostly described as a helicase in genome annotations; however, its predicted 3D structure is related not to the classical DNA unwinding enzymes but to eukaryotic chromatin remodeling proteins such as Ino80 and CHD1L, which are DNA translocases able to slide DNA-bound nucleosomes [66]. While the interactions between these proteins remain obscure, together they may participate in the initiation and propagation of replication forks. The mechanism by which bidirectional replication transitions to concatemer formation remains unclear; however, the encoded Holliday junction resolvase (PBanzai_057) could either facilitate this switch [67] or resolve aberrant DNA structures generated by recombination or stalled replication forks.

No predicted antibiotic resistance of virulence factor genes was found in the genome. The genome also contains no integrase or C repressor genes, which are hallmarks of a lysogenic lifestyle [68]. Additionally, we obtained no hits in a BLASTn search of the terminase large subunit gene in complete bacterial genomes in the NCBI core_nt database, indicating a lack of related prophages.

Recent studies revealed numerous anti-defense genes in *Pbunavirus* genomes that protect them against cellular defense systems [56,69]. The phage Banzai genome encodes for a broad defense inhibitor named Bdi1 (PBanzai_087), protecting from Zorya type I, RADAR, Hypnos and Druantia type I. On the contrary, the neighboring defense gene encoding for protein Bdi2 with similar activity is disrupted by frameshift. Read mapping confirmed that this frameshift is a stable feature, with no detectable heterogeneity within the sample. The genome also harbors genes of TadIII (PBanzai_105) and DadIII-1 (PBanzai_071rev), protecting from Thoeris type III and Druantia type III, respectively. Notably, the latter gene overlaps on the opposed strand with more than half of the sequence of another gene, PBanzai_071, which encodes a protein containing two homologous soluble beta-barrel domains. This protein exhibits higher predicted structural confidence (pTM and pIDDT) in AlphaFold3 compared to DadIII-1 and is more conserved (present in all 45 tested *Pbunavirus* species versus only 29), hinting that DadIII-1 possibly appeared later than overlapping PBanzai_071 via de novo gene birth. Finally, there is a gene encoding ZadI-1 (PBanzai_097) that inhibits both Zorya type I and END nucleases [56,69]. The nearest gene (PBanzai_098) encodes a small protein whose fold is similar to the N-terminal domain of anti-CRISPR protein AcrF2 (PDB 5yhr, RMSD 2.08 Å).

**Figure 7:**
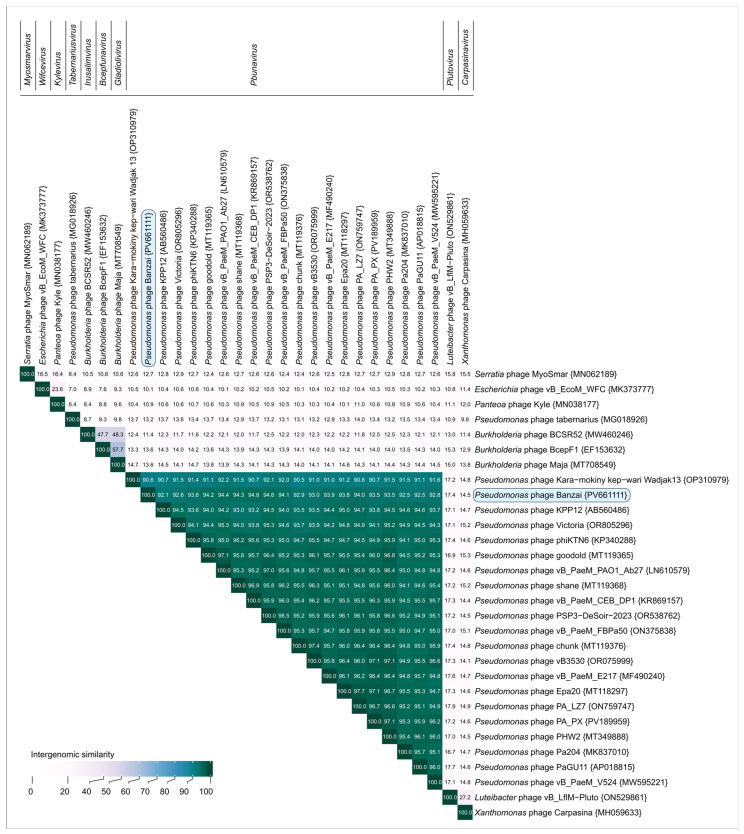
VIRIDIC heatmap generated using genomic sequences of the closest related *Pbunavirus* phages and representative members of other genera of *Lindbergviridae*, classified by the ICTV.

A BLASTn search against the core_nt database and VIRIDIC demonstrated that the closest related phage is *Pseudomonas* phage vB_PaeM_CEB_DP1 (Figure 7), a member of the *Pbunavirus DP1* species according to the ICTV Taxonomy 2024 Release. These phages share 94.8% intergenomic similarity, less than the 95% proposed as the threshold for delineation of phage species. Thus, we believe *Pseudomonas* phage Banzai represents a novel species within the genus *Pbunavirus* according to current recommendations. Noteworthy, when representative members of all 45 *Pbunavirus* species classified by ICTV were compared using VIRIDIC, nearly all the intergenomic similarities were 84.9% or higher, indicating a low diversity within the clade (compared to the 70% similarity cut-off for phage genera). Only two species with shorter genomes due to deletions in structural gene regions (*Pbunavirus datas* and *Pbunavirus FBPa35*) had some values lower.

**Figure 8:**
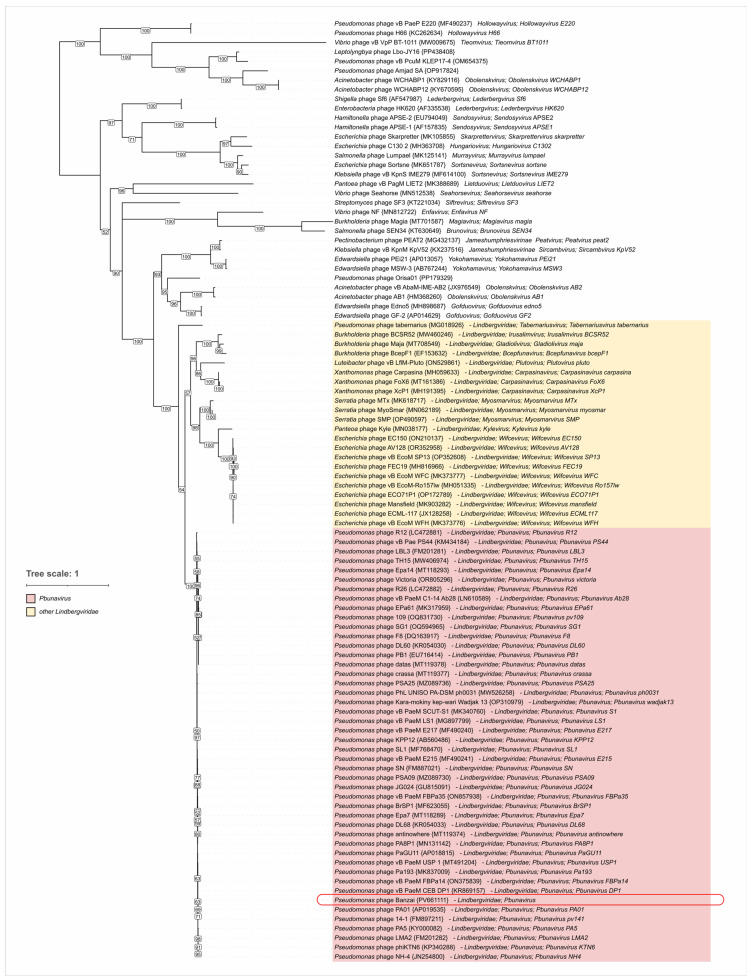
Maximum likelihood phylogenetic tree based on amino acid sequences of the terminase large subunit. Taxonomy is indicated in labels. Nodes with bootstrap values lower than 50% are deleted. The scale bar represents the expected number of substitutions per site. The tree is rooted at the midpoint.

**Figure 9:**
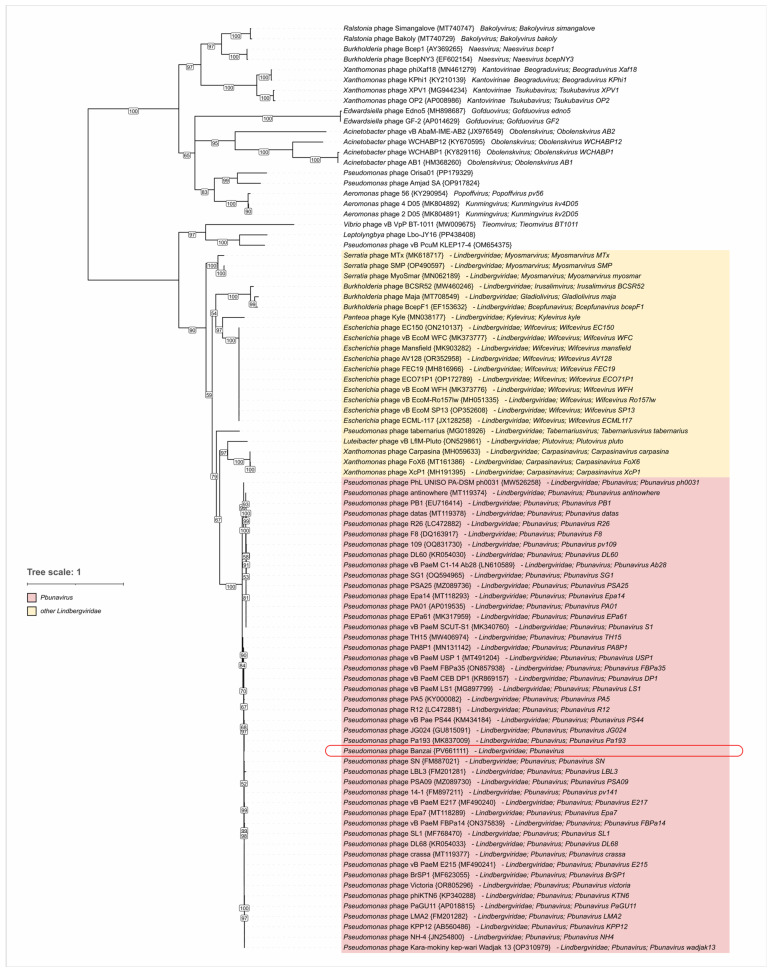
Maximum likelihood phylogenetic tree based on amino acid sequences of major capsid protein. Taxonomy is indicated in labels. Nodes with bootstrap values lower than 50% are deleted. The scale bar represents the expected number of substitutions per site. The tree is rooted at the midpoint.

Additionally, we performed phylogenetic inference of terminase large subunit (TLS) and major capsid proteins (MCPs). Sequences were obtained using tBLASTn searches against a total Genbank *Caudoviricetes* database (E-value < 0.05). The trees (Figure 8 and Figure 9) placed *Pseudomonas* phage Banzai in a clade common with other *Pbunavirus* members, consistent with the results of intergenomic similarity calculations. Inferred clustering demonstrated no contradictions with the *Lindbergviridae* genera included in the ICTV Taxonomy 2024 Release, with all the genera clearly separated. Distantly related homologs were found in various phages mostly infecting *Pseudomonadota*, e.g., the genus *Obolenskvirus*, hinting at possible higher-level taxon grouping. However, this grouping can be undermined by the pervasive horizontal gene transfer. For example, several members of the *Hollowayvirus* genus, including *Hollowayvirus H66* and *Hollowayvirus PA8*, contain genes of *Pbunavirus* TLS homologs; in contrast, *Hollowayvirus phiC725A* and *Hollowayvirus F116* encode an unrelated terminase, indicating that nonhomologous gene replacements have occurred in the evolutionary history of these temperate phages.

## 4. Discussion

### 4.1. Therapeutic Potential of Phage Banzai for Pseudomonas Infections

Pbunaviruses constitute a prevalent group of phages infecting *Pseudomonas* species. Currently, 227 genomes are classified as *Pbunavirus*, with at least 185 isolated using *P. aeruginosa* as the host. Given their lytic lifestyle, activity against *P. aeruginosa*, and lack of antibiotic resistance or virulence factor genes, pbunaviruses represent promising candidates for phage therapy. Generally, they exhibit a broader host range on clinical isolates of *P. aeruginosa* compared to other phage genera [70,71]. However, this result may depend on the strain sampling: in a study using antibiotic-resistant strains of high-risk clones provided by the French National Reference Center for Antibiotic Resistance, pbunaviruses demonstrated lower efficacy compared to other clades, particularly jumbo phages [72]. These conflicting findings highlight the need to identify key susceptibility determinants to adapt phage cocktail formulations for a specific case.

Mutational studies and competitive inhibition assays demonstrated that pbunaviruses use O-specific antigen repeats of lipopolysaccharide for cell adsorption [33,73], unlike certain *P. aeruginosa* phages that exploit type IV pili as receptors (e.g., *Mesyanzhinovviridae* [25]). The binding site does not appear to include the conserved lipopolysaccharide core, as the truncation to the “core + 1 repeat” structure abolishes infection [33,73]. *P. aeruginosa* species comprises 20 serotypes with distinct oligosaccharide structures of O-specific antigen repeats [74,75], and genomic studies revealed loci architectures allowing to distinguish 14 serogroups, with each referring to one to three serotypes [54,75]. Our study demonstrated that *Pseudomonas* phage Banzai successfully propagates in members of serogroups O1, O3, O5 and O6, but not in the widespread O11 serogroup. It is currently unknown how its tail fiber protein successfully binds such a diverse array of polysaccharide structures.

To date, systematic studies on the association between serogroup, sensitivity to *Pbunavirus* phages and their tail fiber protein sequences are lacking. However, it has been shown that different pbunaviruses vary in their ability to adsorb to the cell surface of different strains of *P. aeruginosa* [71]. While phage Banzai was incapable of infecting serogroup O11 strains, the ability of at least one *Pbunavirus* phage (MX331) to lyse them has been previously described [72]. Adaptation to different host populations with diverse surface receptors explains the high variability and mosaic sequence of tail fiber proteins in their C-terminal regions, which putatively participate in O-specific antigen binding. Resistance of some strains within the infected serogroups can be explained both by specialized phage defense systems and loss of O-antigen expression, which is common among cystic fibrosis strains adapted to chronic infection [76].

One mechanism by which *Pseudomonas aeruginosa* acquires heightened antibiotic tolerance is biofilm formation, whereby bacteria organize into structured communities embedded in an extracellular polymeric matrix [77]. Although the present study focuses on a different aspect of *Pbunavirus* biology, any effective therapeutic strategy against *P. aeruginosa* could ultimately integrate agents that couple direct antibacterial activity with the disruption or prevention of biofilm architecture [78]. *Pbunavirus* phages prevent biofilm formation and directly lyse biofilm-embedded bacteria, reducing biofilm biomass and enhancing the efficacy of antibiotics by lowering the minimum inhibitory and bactericidal concentrations required to kill biofilm-associated cells [26,79,80]. Despite the lack of phage-encoded depolymerase enzymes, they are hypothesized to induce the expression of the cell’s own hydrolytic enzymes [26].

As such, *Pseudomonas* phage Banzai is a strong candidate for incorporation into phage cocktail formulations and personalized phage therapy regimens to target non-O11 multidrug-resistant *Pseudomonas aeruginosa*. While Banzai’s therapeutic capabilities warrant further investigation, our in vitro stability assays demonstrated relatively high stability under physiological conditions (37 °C and pH 7). Moreover, in vivo experiments using the *G. mellonella* model showed significant improvement in larval survival after 24 h following phage Banzai treatment.

### 4.2. General Virion and Genome Features

The overall genome architecture of *Pbunavirus* is highly conserved. The terminase, essential for packaging, is represented by large and small subunits (PBanzai_001 and PBanzai_081, respectively), with their genes separated as reported for phage E217 [81]. The main co-oriented gene cluster encodes structural proteins (PBanzai_018–PBanzai_048). This region exhibits synteny and high sequence identity (>96%) with homologous regions from *Pbunavirus* phages Pa193 and E217 studied by cryo-electron microscopy [32,33]. Based on these findings, we infer that these phages share a similar virion structure, including a capsid-surrounding “cage” formed by a decoration protein and a simple baseplate.

An interesting observation is that *Pseudomonas* phage Banzai’s genome exhibits a guanine–cytosine (GC) content of 55.5%, notably lower than the ~67% observed in *P. aeruginosa* [27,82]. This divergence in GC content could be attributed to *Pbunavirus*’s own replication machinery, comprising DNA polymerase, primase, and helicases. Alternatively, the primary natural hosts for *Pbunavirus* phages might be *Pseudomonas* species with lower GC content, whose representatives are currently underrepresented in phage isolation efforts due to the clinical focus on *P. aeruginosa*. While a previous study demonstrated a broad host range for four *Pbunavirus* phages, encompassing *Escherichia*, *Chryseobacterium*, and *Microbacterium* [83], the vast majority of *Pbunavirus* genomes annotated with specific hosts in GenBank (e.g., using “/host” or “/lab_host” qualifiers) are exclusively associated with P. aeruginosa, with a few exceptions in *P. syringae*. Though no *Pbunavirus*-related prophages have been identified in circular bacterial genomes, some draft assemblies of non-*Pseudomonas* species do contain *Pbunavirus* contigs (e.g., NZ_JAESED010000097.1, NZ_JABTZZ010000019.1). However, we interpret these findings as potential contamination rather than evidence of viral replication in these species. Thus, current data do not support widespread infection of other bacterial species by *Pbunavirus* phages at levels sufficient to significantly influence the observed GC content.

### 4.3. Genome Packaging

Prior reports on *Pbunavirus* genome termini have been contradictory. Early studies suggested that PB1-like phages possess defined physical genome ends, based on restriction enzyme digestion patterns [82]. However, subsequent research on *Pseudomonas* phage JG024 argued for a circularly permuted linear genome, based on read coverage analysis and amplification of overlapping fragments [57]. Our study supports a headful packaging mechanism, resulting in a circularly permuted genome. This characteristic should be inherent to all *Pbunaviruses* due to their closely related terminase and other conserved packaging machinery components.

Phages utilizing headful packaging like Banzai are prone to generalized transduction, enabling the transfer of nearly any fragment of the host genome from one cell to another [84,85]. This mechanism arises because these phages need only recognize a single *pac*-like site to initiate chromosomal packaging, proceeding to fill the capsids until the terminase complex disengages from the chromosome [84]. Presumably, this process occurs in phages with circularly permuted genomes that do not degrade the host DNA during lytic infection [85]. There is evidence from the pre-genomic era that mutant and wild-type pbunaviruses are indeed capable of generalized transduction [86]. The natural contribution of this process, carried out by *Pbunavirus*, to the genomic variability of *P. aeruginosa* remains unclear, but it is intriguing to hypothesize that these parasites, while possessing a stable genome organization themselves, provide their hosts with evolutionary flexibility. However, this imposes additional constraints on the selection of the production strain for obtaining therapeutic phage preparations: it should contain a minimum of beneficial loci that could be transferred from it to the pathogen.

### 4.4. Lateral Gene Transfer and Genomic Evolution Within Pbunavirus

Phylogenetic analysis based on major capsid protein and terminase large subunit sequences from phage Banzai and related phages reveals both conserved and divergent evolutionary patterns. Both trees support the monophyly of the *Lindbergviridae* family and the genus *Pbunavirus*. Notably, the trees also indicate affiliations with several groups of lytic phages, including members of the *Obolenskvirus* genus [87,88]. *Acinetobacter baumannii* (the host of *Obolenskvirus*) and *P. aeruginosa*, despite their long evolutionary distance, share many common ecological traits. They are not only clinically relevant nosocomial pathogens causing ventilator-associated pneumonia and other infections [89] but also ubiquitous saprophytes found in soil and water [90,91]. We suppose that the tree topology both within *Lindbergviridae* and outside of this clade reflects the natural history of phage host switches between predominantly free-living bacteria facilitated by shared ecological niches or even the ability to form multi-species biofilms as previously shown for *P. aeruginosa* and *Burkholderia cepacia* [92]. Initially accidental infection of non-native neighboring host species, supported by natural selection, can drive phage lineage diversification, reducing competition between related phages due to niche partitioning and serving as a protection from collapse of the primary host population.

However, the divergent composition of neighboring branches in the MCP and TLS trees suggests a complex evolutionary history involving genetic exchange events preceding the emergence of *Lindbergviridae* and *Pbunavirus*. This underscores the potential for lateral gene transfer (LGT) early in their evolution.

Ceyssens et al. previously reported limited LGT in *Pbunavirus* phages, highlighting a conserved core genome region and absence of large-scale acquisition of novel regions or functionally equivalent gene replacements [82]. This conservation suggests that virion structure and replication mechanisms are largely shared among *Pbunavirus* phages, including phage Banzai. This may be explained by constraints from protein–protein interactions within virion particles and replication complexes [82], coupled with limited capsid capacity. We speculate that the “cage” of decoration proteins [32] may act as an additional physical constraint limiting head size variation and thereby restricting genetic cargo.

Alternatively, the limited structural diversity in *Pbunavirus* phages might reflect their relatively recent origin. The observed diversity in nucleotide sequences, conversely, may stem from a high nucleotide substitution rate during low-fidelity replication by phage polymerase. This hypothesis is supported by observations of significant *Pbunavirus* microdiversity even within a single ecological niche [83]. However, this contradicts the presence of a proofreading exonuclease in the phage replication machinery, which might mitigate high error rates. Finally, the strictly virulent lifestyle of *Pbunavirus* phages could contribute to this pattern: unlike temperate phages (e.g., Lambdoid phages [93]), *Pbunavirus* phages cannot encounter closely related prophages within the host genome to facilitate recombination.

## 5. Conclusions

*Pseudomonas* phage Banzai, a lytic phage active against a substantial number of isolates tested, warrants consideration for inclusion in phage cocktails or personalized phage therapy strategies to target non-O11 *Pseudomonas aeruginosa* strains. Based on ICTV criteria, phage Banzai, a member of the *Pbunavirus* genus within the *Lindbergviridae* family, represents a novel species. The phage genome, comprising 66,189 bp and encoding 105 putative proteins, exhibits a significantly lower GC content compared to its *P. aeruginosa* host. The genome lacks predicted antibiotic resistance or virulence factor genes and indicates a strictly lytic life cycle. Genome and proteome analyses revealed evolutionary relationships with the *Obolenskvirus* and *Gofduovirus* genera and suggest that phage adsorption involves O-antigen polysaccharide recognition.

## Data Availability

All relevant data are available within this article and its Supplementary Materials. The *Pseudomonas* phage Banzai genome sequence is deposited in NCBI GenBank under accession number PV661111.

## Supplementary Materials

The following supporting information can be downloaded at https://www.mdpi.com/article/10.3390/v17081088/s1, Table S1: Sensitivity of *Pseudomonas aeruginosa* isolates to *Pseudomonas* phage Banzai.

## Abbreviations

The following abbreviations are used in this manuscript:

bp	base pair
LGT	lateral gene transfer
MCP	major capsid protein
TLS	terminase large subunit
