# Supplementary material for: Pseudomonas Phage Banzai: Genomic and Functional Analysis of Novel Pbunavirus with Lytic Activity Against Pseudomonas aeruginosa
Source: Viruses. 2025 Aug 6;17(8):1088. doi: 10.3390/v17081088 (PMC12390725; doi:10.3390/v17081088)
Supplement: Supplementary file 1 [file viruses-17-01088-s001.zip › Supplementary_materials.pdf]

Article

Supplementary Materials

Table S1. Sensitivity of *Pseudomonas aeruginosa* isolates to *Pseudomonas* phage Banzai. ST, sequence type determined using PubMLST; ND, no data; NT, non-typeable.

P. aeruginosa isolate	ST	Sensitivity to bacteriophage (++ Pronounced lysis, + Lysis and – No lysis observed)	In silico predicted serogroup (included serotypes)
PAO1 laboratory Krylov	549	++	O5 (O5, O18, O20)
PAO1	549	++	O5 (O5, O18, O20)
97-1	358	+	O5 (O5, O18, O20)
99-1	3765	++	O6 (O6)
158-4	2465	+	O6 (O6)
189-1	274	+	O3 (O3)
220/2	274	-	O3 (O3)
233-2	245	-	O5 (O5, O18, O20)
105/4	200	++	O3 (O3)
17902-1	635	-	O6 (O6)
17905-1	2592	-	O11 (O11)
17911-2	1205	-	O6 (O6)
17(2)	231	+	O6 (O6)
62(1)	3496	-	O6 (O6)
80/2	233	-	O6 (O6)
82/2	254	++	O1 (O1)
82/2_mucoid	ND	+	ND
98/3	235	-	O11 (O11)
128/2	235	-	O11 (O11)
215/4	308	-	O11 (O11)
239/1	242	+	NT (derived from O1)
294 I	1203	-	O5 (O5, O18, O20)
294 I_mucoid	ND	-	ND
294 II	1203	-	O2 (O2, O16)
316 I	235	-	O11 (O11)
316 II	235	-	O11 (O11)
317	235	-	O11 (O11)
325 I	12	++	O5 (O5, O18, O20)
17903	395	++	O6 (O6)
41748/2	235	-	O11 (O11)
